# Tick-borne pathogens *Ehrlichia*, *Hepatozoon*, and *Babesia* co-infection in owned dogs in Central Thailand

**DOI:** 10.3389/fvets.2024.1341254

**Published:** 2024-04-02

**Authors:** Tatiyanuch Chamsai, Aeknarin Saechin, Chalisa Mongkolphan, Ladawan Sariya, Siriporn Tangsudjai

**Affiliations:** Monitoring and Surveillance Center for Zoonotic Diseases in Wildlife and Exotic Animals, Faculty of Veterinary Science, Mahidol University, Nakhon Pathom, Thailand

**Keywords:** co-infection, *Ehrlichia*, *Hepatozoon*, *Babesia*, prevention of tick-borne diseases

## Abstract

Tick-borne pathogens are transmitted by a wide range of tick species and affect both human and animal health. Understanding the diversity of these pathogens and their co-infection rates in domesticated animals in urban areas is crucial for effective disease management and prevention. In this study, a total of 565 owned dogs in the central region of Thailand were investigated for the infection rate of three genera of *Ehrlichia*, *Hepatozoon*, and *Babesia* infection using multiplex PCR. The results revealed an overall infection rate of 19.1%, with *Ehrlichia* having the highest infection rate (12.2%), followed by *Babesia* (2.5%) and *Hepatozoon* (1.4%). The rate of co-infection was 3%, with mixed infections involving two or three genera. Male dogs exhibited a slightly higher infection rate compared to females, although not statistically significant. Young adult dogs (1–3 years) showed the highest infection rate of both single infections and co-infections. Monthly infection rate indicated variations throughout the year, with co-infection rate significantly associated with overall infection rate. Clinical manifestations in three genera of infected dogs included thrombocytopenia and eosinopenia. The results of this study are useful to design strategies for the management and prevention of tick-borne diseases in the study area.

## Introduction

Tick-borne diseases in dogs are caused by various etiologic agents that are transmitted to dogs from infected tick. The common tick-borne pathogens that affect dog health include *Ehrlichia* spp., *Anaplasma* spp., *Hepatozoon* spp., and *Babesia* spp. *Ehrlichia* is an intracellular bacterium causing canine monocytic ehrlichiosis. Dogs infected with *Ehrlichia* can develop a range of clinical signs, including fever, loss of appetite, weight loss, joint pain, enlarged lymph nodes, anemia, and bleeding disorders (1). It can be a serious and potentially life-threatening disease. *Ehrlichia* is transmitted to dogs through saliva of the brown dog tick (*Rhipicephalus sanguineus*) (2). *Hepatozoon* is a protozoan parasites. *Hepatozoon canis* (*H. canis*) has been reported to infect dogs across inhabited continents (3–6). It is transmitted by ingesting an infected tick (*Rhipicephalus sanguineus* and possibly other tick species) or by ingesting an animal that contains the larval stages (7). *Hepatozoon* infections can range from no apparent to severe clinical manifestation, although dogs are most often subclinically or mildly affected. Dogs with compromised immune systems are more likely to develop more severe forms of the disease (8). Clinical signs may include fever, anemia, lethargy, and anorexia, which are commonly observed. Canine babesiosis is caused by an intraerythrocytic protozoan belonging to the *Babesia* genus. In dogs, *Babesia canis* (*B. canis*) (subspecies: *canis*, *vogeli*, and *rossi*), *B. gibsoni*, *B. conradae*, and *B. microti* have been identified (9). *Babesia* is widely distributed, making it a significant concern for animal health worldwide (10, 11). The geographical distribution of each *Babesia* spp. varies (9). *Babesia* is transmitted through the biting of hard tick vector (Ixodidae) species. Infected dogs typically lack obvious clinical symptoms. Clinical manifestation differed depending on the strain of *Babesia* involved (12). However, all strains could develop hemolysis, thrombocytopenia, and hyperglobulinemia.

Tick-borne disease infections are most commonly associated with warm and humid regions. In endemic areas, infection occurs throughout the year. The prevalence of tick-borne infections in adult dogs is higher than in younger dogs (13). Dogs over 3 years old were reported as a factor associated with *Ehrlichia canis* (*E. canis*) infection in dogs (14). Dogs exhibiting thrombocytopenia along with other clinical signs of tick-borne disease were more likely to be infected by tick-borne disease pathogens (15). Co-infection of multiple tick-borne pathogens in dogs has been reported. There is a significant association between exposure to *Anaplasma* spp. and *Babesia* spp. in the United States, with 36% of hunting dogs exposed to *Anaplasma* species infection having been exposed to *Babesia* spp. (16). Co-infection of *E. canis* and *H. canis* or *B. canis* and *H. canis* has also been detected in dogs, and they exhibit clinical signs like thrombocytopenia, leukopenia, fever, or lethargy (17). Co-infection with tick-borne pathogens can occur through a single tick that carries multiple pathogens. There have been reports of multiple tick-borne pathogens in individual ticks, with reported rates ranging from 1% to over 10%, with more than one pathogen present (18, 19). The study aims to provide valuable insights into the infection rate, co-infection patterns, risk factors, and potential hematological alterations of three genera of tick-borne pathogens, *Ehrlichia*, *Hepatozoon*, and *Babesia*, in dogs in the central region of Thailand.

## Materials and methods

### Study design and subject selection

The present study was carried out using the data collected during January to December 2022 on 565 owned dogs that were suspected to be suffering from various tick-borne diseases and treated at Prasu Arthorn Veterinary Teaching Hospital of the Faculty of Veterinary Science, Mahidol University, Thailand. These dogs were investigated for the presence of multiple tick-borne pathogens including *Ehrlichia*, *Hepatozoon*, and *Babesia*, complete blood count (CBC), and blood biochemistry. The tick-borne pathogen detection results of 565 dogs, which are 299 males and 266 females, were gathered from the Monitoring and Surveillance Center for Zoonotic Diseases in Wildlife and Exotic Animals (MoZWE). The CBC and blood biochemistry data of positive tick-borne pathogen dogs were collected from Prasu Arthorn Veterinary Teaching Hospital. All owners completed a consent form authorizing the use of data. In the study of infection rate by age, only 530 dogs could be identified by birth date and were categorized into five age groups: puppies (less than 1 year); young adults (1–3 years); adults (4–6 years); old (7–10 years); and very old (>10 years).

### Blood DNA extraction and multiplex-PCR

Two hundred microliters of blood were used to extract DNA using the genomic DNA mini kit^®^ (Geneaid, Taipei, Taiwan). Samples were incubated in lysis buffer (0.1 M NaCl, 10 mM Tris-Cl (pH 8.0), 5% SDS) with 10 μL of proteinase K (10 mg/mL) (minimum 20 min) at 56°C. Purification of DNA was conducted in accordance with the protocol described by the manufacturer. DNA was dissolved in 50 μL of 10 mM Tris–HCl pH 8.0 containing 1 mM EDTA. DNA was kept at −20°C until investigation. Specific three primer pairs of virB9 protein gene of *Ehrlichia* (forward, Ehr1401F, 5′- CCATAAGCATAGCTG ATAACCCTGTTACAA -3′, and reverse, Ehr1780R, 5′- TGGATAA TAAAACCGTACTATGTATGCTAG -3′) (20), 18S rRNA gene of *Hepatozoon* (forward, Hep001F, 5′- CCTGGCTATACATGAGCAAA ATCTCAACTT -3′, and reverse, Hep737R, 5′- CCAACTGTCC CTATCAATCATTAAAGC -3′) (20), and the 18S rRNA gene of *Babesia* (forward, Ba143-167, 5′- CCGTGCTAATTGTAGGGCTAATACA -3′, and reverse, Ba694-667, 5′- GCTTGAAACACTCTARTTTCTCAAAG -3′) (21) were used for Multiplex PCR amplification. A total volume of 50 μL of reaction consists of 5 μL of template DNA, 25 μL of 1x multiplex PCR master mix (QIAGEN, Germany), and 1 μL of each primer at a concentration of 0.2 μM. A C1000 touch thermal cycler (BIO-RAD, Hercules, CA, USA) was used. Amplification conditions were initialed at 95°C 15 min, followed by 35 cycles of 94°C 45 s, 61°C 45 s, 72°C 1 min, and final extension at 72°C 10 min. PCR products were separated on 2.0% agarose gel and stained with GelRed (Bio-tium, Fremont, CA, USA). DNA bands were visualized by UV transluminator. The expected sizes for *Ehrlichia*, *Hepatozoon* and *Babesia* were 380, 737, and 525 bps, respectively.

### Determination of hematological and serum biochemical parameters

Blood (1 mL per sample) was collected from the cephalic veins into tubes containing ethylenediamine tetra-acetic acid-coated tubes. CBC was done on an automated Mindray BC-5300vet hematology analyzer (Mindray, Shenzhen, China) within 2 h of sampling.

Total white blood cell count, differential count of leucocytes, red blood cells, hemoglobin concentration, hematocrit, mean corpuscular volume, mean corpuscular hemoglobin, mean corpuscular hemoglobin concentration, RBC distribution width, and platelets were determined.

Non-hemolysed serum was obtained from the blood samples. This serum was used to determine biochemical parameters using the AU-480 chemistry analyzer (Beckman Coulter, Brea, USA).

The parameters measured included total serum proteins and serum albumin.

### Statistical analyses

The data were analyzed using the SPSS 27 (IBM, Armonk NY, USA). The study compared the infection rate across various categories. Fisher exact tests were used to examine differences between sex groups. The differences between the five age groups were analyzed using Pearson χ^2^. The likelihood-ratio G^2^ was used to assess differences between sampling periods (months). A *p* value of <0.05 was considered statistically significant. Pearson correlation coefficients were determined to assess the correlation between the monthly infection rate of overall infection and co-infection.

## Results

**Figure 1:**
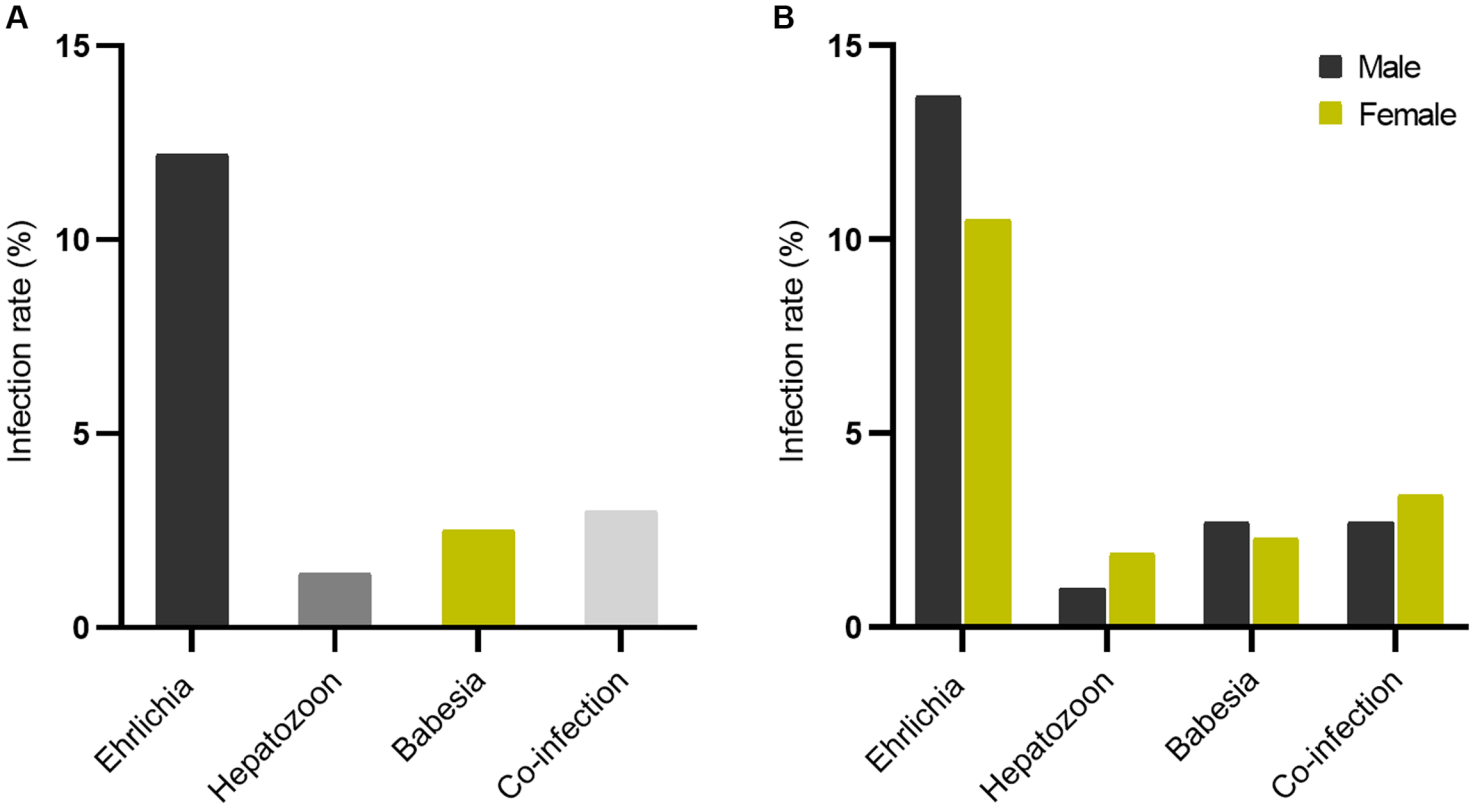
Tick-borne pathogens: *Ehrlichia*, *Hepatozoon*, *Babesia*, and co-infection rate **(A)** and sex-separate infection rate **(B)**.

**Figure 2:**
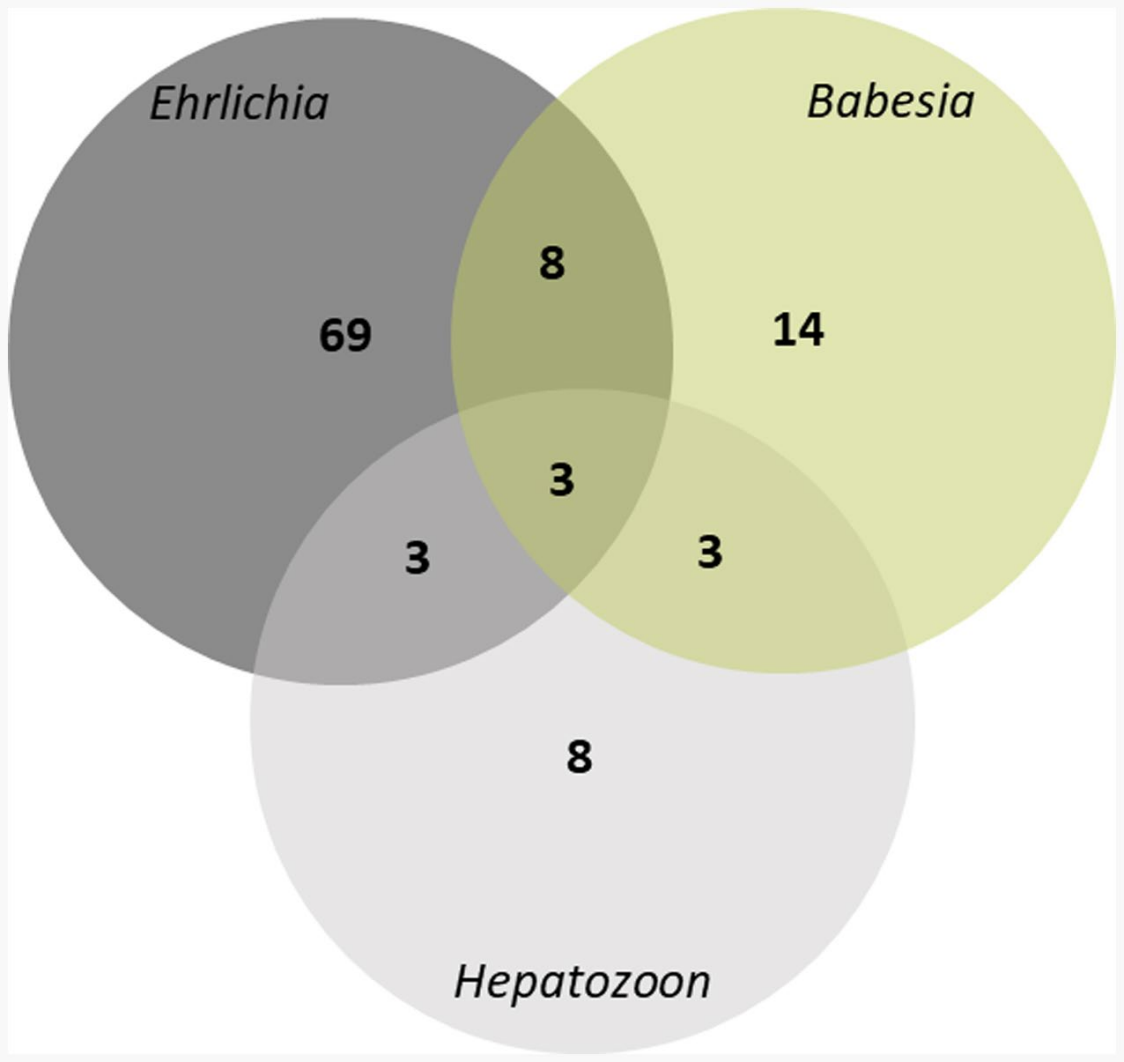
Venn diagram showing the composition of tick-borne pathogens infections, a total of 565 owned dogs were investigated for *Ehrlichia*, *Hepatozoon*, *Babesia* using multiplex PCR.

A total of 565 owned dogs were investigated for *Ehrlichia*, *Hepatozoon*, and *Babesia* using multiplex PCR. 108 (19.1%) samples tested were positive for at least one pathogen. The highest infection rate was *Ehrlichia* infection, 69/565 (12.2%). *Hepatozoon* infection rate was lowest at 8/565 (1.4%). *Babesia* infection rate was 14/565 (2.5%). Co-infection rate, where a dog was positive for more than one pathogen, was 17/565 (3%) (Figure 1A). Co-infection of two or three genera was observed. The mixed infections between *Ehrlichia* and *Hepatozoon*, *Ehrlichia* and *Babesia*, and *Hepatozoon* and *Babesia* were found in 3, 8, and 3 samples, respectively. Co-infection involving *Ehrlichia*, *Hepatozoon*, and *Babesia* was detected in 3 samples (Figure 2).

**Figure 3:**
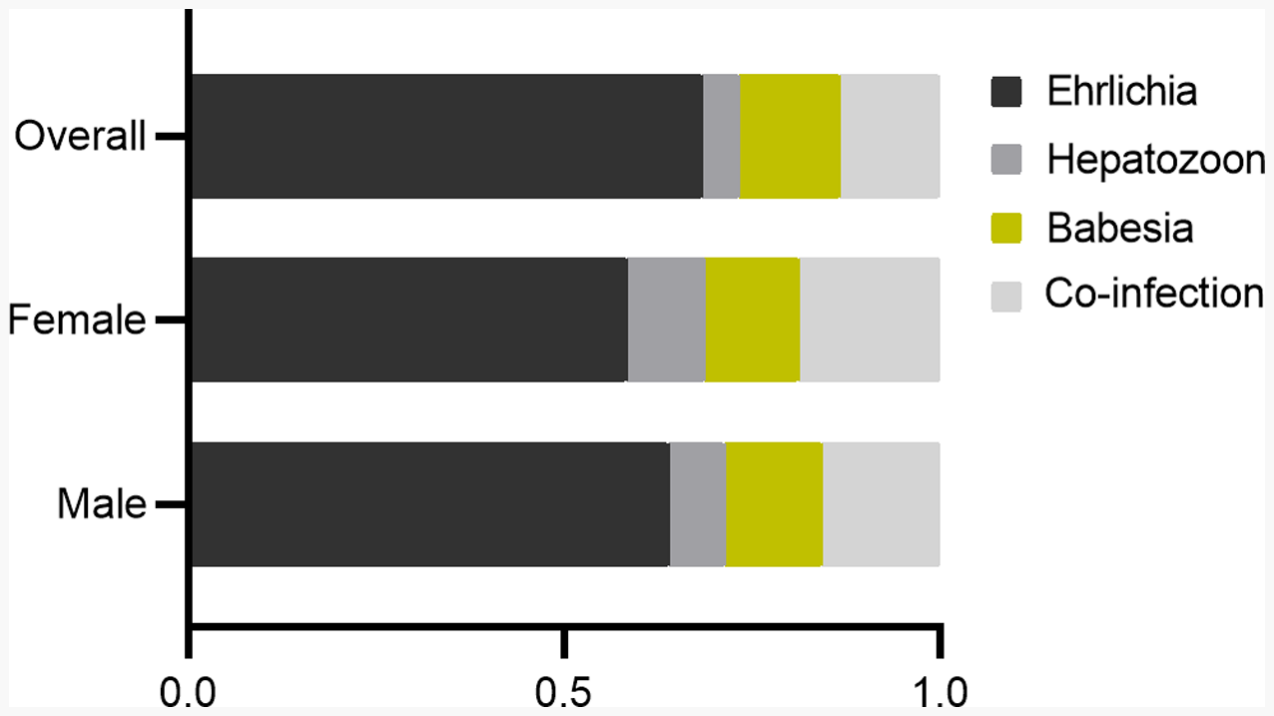
Bar chart showing the proportions of *Ehrlichia*, *Hepatozoon*, *Babesia*, and co-infection infected in male, female, and overall populations (based on infection rate data).

**Figure 4:**
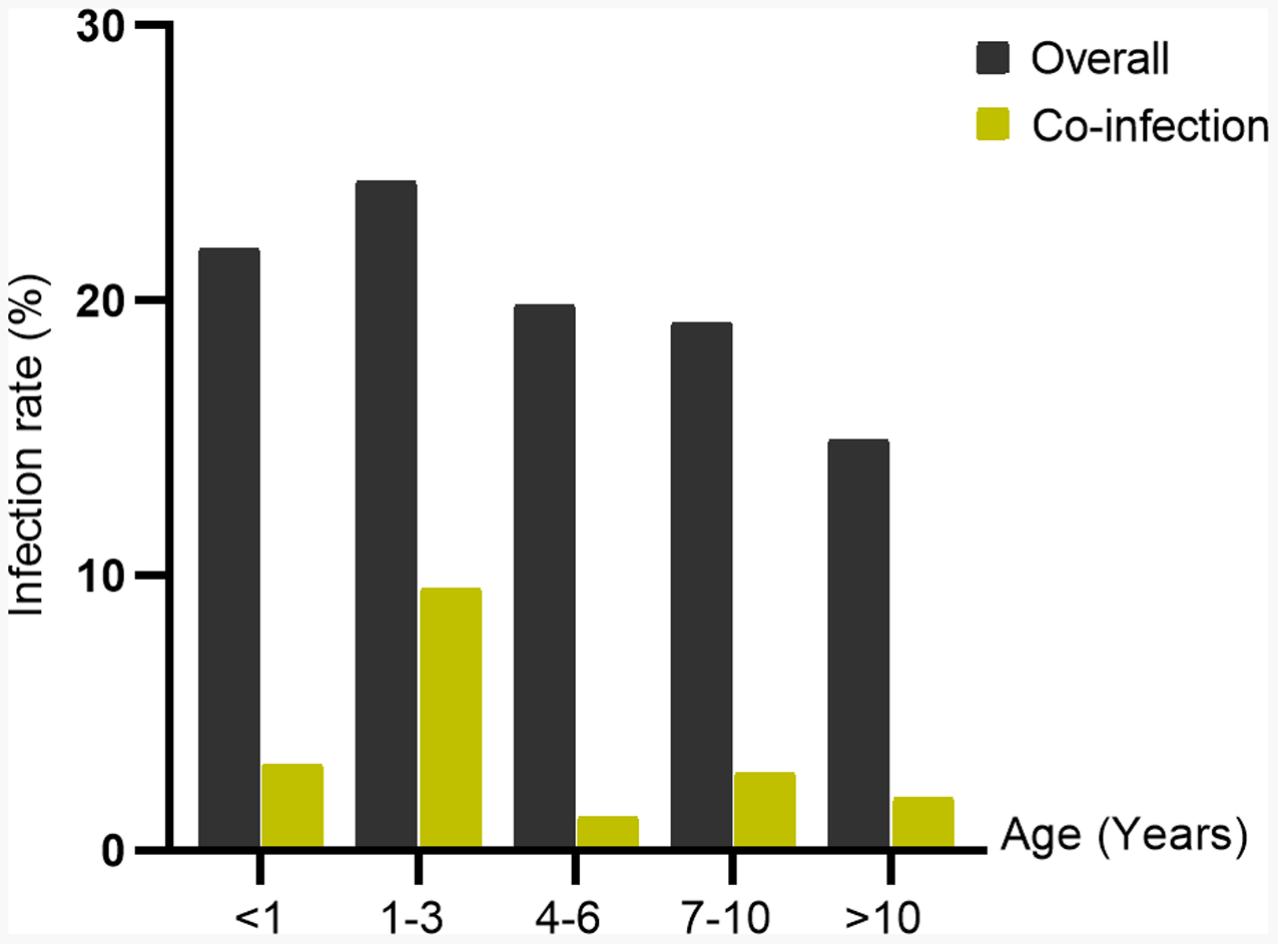
Bar chart demonstrating the infection rate of tick-borne pathogens: overall and co-infection by age group.

**Figure 5:**
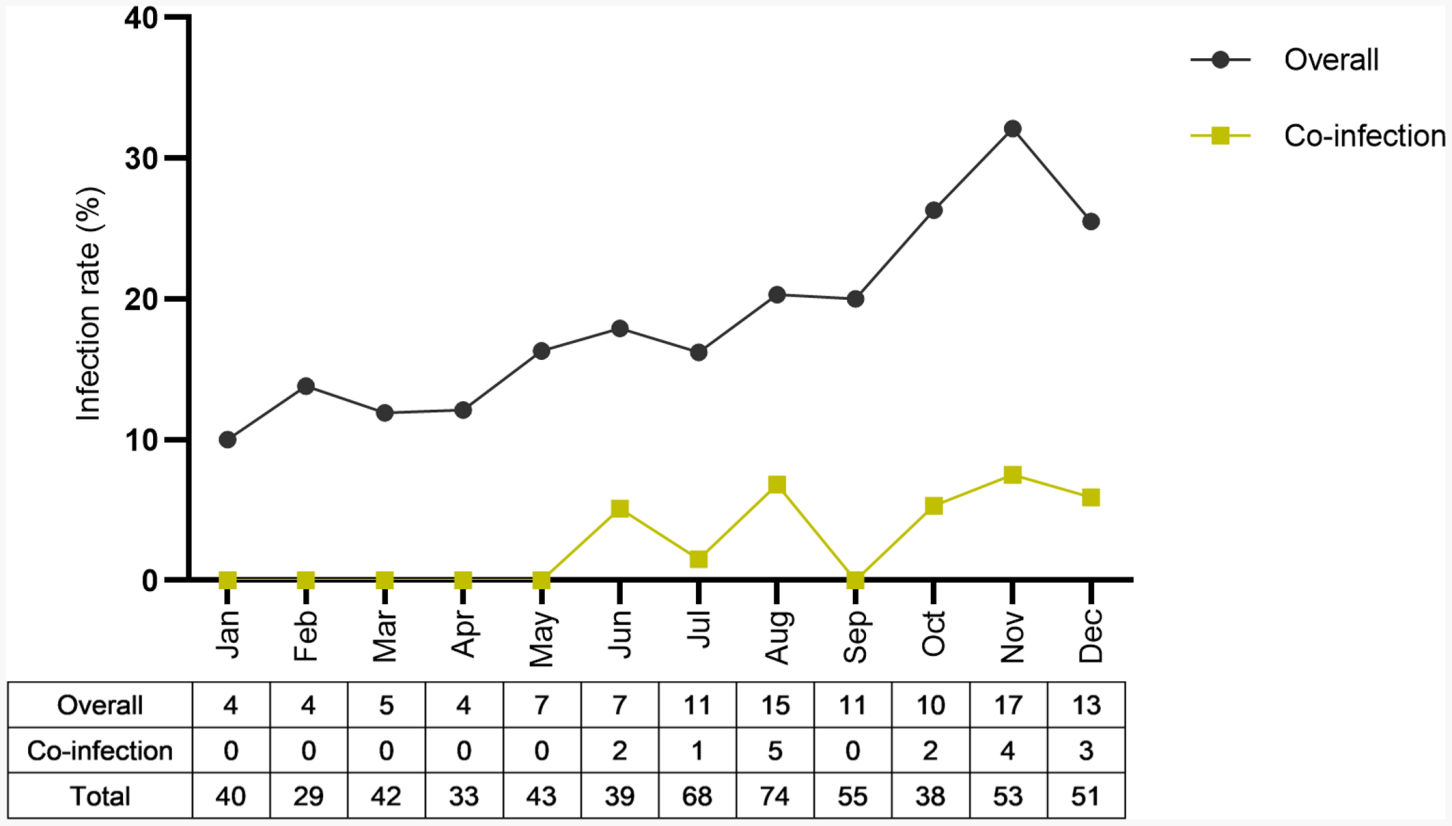
Annual infection rate throughout the year (2022) was presented. Overall and co-infection monthly infection rate was reported as a seasonal trend pattern.

Overall infection rate by sex showed that the infection rate was higher in male dogs (60/299, 20.1%) as compared to female dogs (48/266, 18.0%), but not statistically significant (*p* = 0.592) (Supplementary Table 1A). The sex difference in infection rate is presented in Figure 1B. The proportion infected by sex is represented in Figure 3. Both sexes had a high infection rate of Ehrlichia, with male dogs exhibiting a higher infection rate than female dogs. Female dogs showed a higher infection rate of Hepatozoon and co-infection than male dogs. The infection rate of Babesia in male dogs was higher than in female dogs. There were no statistically significant differences between sexes in infection rate in all three genera of infection and co-infection (Supplementary Table 1A). Infection rate by age: five age groups were presented (Figure 4). The young adult age group (1–3 years) exhibits the highest infection rate both of overall infection rate (18/74, 24.3%) and co-infection rate (7/74, 9.5%) (Supplementary Table 1B). The co-infection rate was significantly different between age groups (*p* = 0.022). The infection rate of tick-borne pathogens changes over the course of a year. The monthly infection rate showed that Ehrlichia can be infect throughout the year (Supplementary Table 2). The increase of overall and co-infection rates occurred from June to December (Figure 5). The difference between infection rates by months was significant in co-infection but not overall (*p* = 0.018, *p* = 0.232 respectively). Moreover, co-infection rate is associated with overall infection rate (Pearson’s *r* = 0.81, *p* < 0.001).

The blood alterations in *Ehrlichia* infected dogs were thrombocytopenia (57/66), eosinopenia (45/66), hyperproteinemia (43/66), and anemia (23/66). Dogs with *Hepatozoon* infection had prominent thrombocytopenia (5/7) and hyperproteinemia (4/7). Hematological changes in dogs infected with *Babesia* were thrombocytopenia (10/11), eosinopenia (9/11), hyperproteinemia (5/11), and anemia (5/11). The hematological alterations in co-infection dogs were observed. All three dogs co-infected with *Ehrlichia*, *Hepatozoon*, and *Babesia* developed leukocytosis (3/3), thrombocytopenia (3/3), eosinopenia (3/3), and hyperproteinemia (2/3) (Supplementary Table 3).

## Discussion

The risk of disease transmission from ticks varies based on the geographic region and the specific tick species present there. Hong Kong indicates that the prevalence of *Babesia* spp. (28.8%) was higher than *E. canis* (7.4%) (22). In an Ethiopian study of tick-borne infections in dogs, *H. canis* registered the highest frequency of occurrence (53.8%), with *E. canis* infection determined at 2.6% (5). Our finding found the highest infection rate was *Ehrlichia* infection (12.2%). The difference in infection rates for the predominant pathogen may be due to various factors, including tick species, climate and ecology, frequency of tick-host interactions, and control measures (23, 24). Understanding the specific tick species and pathogens present is crucial for effective disease prevention and control. However, our study did not determine the infection rate of pathogens in dog ticks, which is an area that requires further investigation and should be addressed. The study in Northeast Thailand showed the high prevalence of *H. canis* (65.71%), followed by *Babesia* spp. (31.43%) and *E. canis* (30.0%), in brown dog ticks from infected dogs (25). In contrast, another research in same area found that *E. canis* is the most single pathogen in dogs (64.0%) and ticks (82.0%) (26). It’s essential to consider the possible role of seasonal variation and environmental factors. Our findings illustrate that *Ehrlichia* infection cases can occur throughout the year. While *Babesia* and *Hepatozoon* appear to be more infected rate during the wet season (Supplementary Table 2). The study suggests that climate change is affecting the distribution and infection rate of tick-borne pathogens, with increased rainfall intensity in May leading to an increase in the infection rate of individual pathogens and co-infections (Supplementary Table 2). The observed patterns in monthly infection rate suggest potential fluctuations in pathogen infection rate over time, but the limited duration of our study may not fully reflect the complexity of seasonal dynamics. Future research efforts might benefit from extending the study period across several years in order to completely analyze seasonal fluctuations in tick-borne pathogen co-infection occurrence.

This study aimed to identify tick-borne pathogens in dogs of different ages and allow a comprehensive understanding of age-related variations in these infections. Our findings reveal that co-infection can occur in any age group. However, co-infection of *Ehrlichia*, *Hepatozoon*, and *Babesia* was detected in dogs under 3 years. Dogs aged between 1and 3 years had the highest overall and co-infection rate. This suggests that younger age group is more susceptible to these infections compared to other age groups. The study in Costa Rica identified that the age of dogs, between 2 and 7 years, significantly influences the risk factors associated with *E. canis* seropositivity (27). Young age, less than 1 year, was identified as a risk factor for the co-infection prevalence of tick-borne pathogens in South India (28). The difference between susceptible age groups could be due to various factors, such as regional differences or variations in the study populations. Our findings emphasize that the age of dogs can play a significant role in their susceptibility to tick-borne infections and co-infections. Understanding these age-related variations is essential for effective prevention and management of tick-borne diseases in dogs.

Multiple infections caused by tick-borne pathogens are common in tropical and endemic areas where vectors are abundant. *Rhipicephalus sanguineus* is the main vector of *E. canis*, *H. canis*, and *B. canis* with different transmission route. Concurrent infections can have significant implications for disease pathogenesis and clinical symptoms. Co-infection has been reported to cause hematological alterations, including severe thrombocytopenia, anemia, and hypoalbuminemia (29). Another study through clinical pathological changes in co-infection of tick-borne pathogens in dogs identified anemia, thrombocytopenia, hypoalbuminemia, increased β2 and γ globulin fractions, and an increase in C-reactive protein concentrations (30). Our study found that co-infection in dogs can cause an increase in thrombocytopenia (Supplementary Table 3). In addition, leukocytosis, thrombocytopenia, eosinopenia, and hyperproteinemia were observed during the three pathogen infections. The pathogenicity of co-infections requires further investigation of how these co-infections interact and influence each other. Understanding how these co-infections interact with each other is necessary for both diagnosis and treatment.

## Conclusion

The study provides valuable data for disease management and prevention related to tick-borne diseases in urban areas. The study suggests that young adult dogs (aged 1–3 years) had the highest infection rate of both single infections and co-infections. The rate of the co-infection of tick-borne pathogens exhibits an occurrence time interval. Understanding the infection rate and risk factors, such as age and seasonality, is crucial for implementing effective prevention and control measures.

## Data availability statement

The original contributions presented in the study are included in the article/Supplementary material, further inquiries can be directed to the corresponding author.

## Ethics statement

Ethical approval was not required for the studies involving animals in accordance with the local legislation and institutional requirements because this study did not directly use animal samples; it only used data from animals that had previously been treated and diagnosed in an animal hospital. The researcher contacted the animal owners to explain the scope of the research and request permission to use animal data in this research. Written informed consent was obtained from the owners for the participation of their animals in this study.

## Author contributions

TC: Data curation, Writing – review & editing. AS: Conceptualization, Formal analysis, Visualization, Writing – original draft. CM: Investigation, Writing – original draft. LS: Validation, Writing – original draft. ST: Writing – original draft.
