# Supplementary material for: Tick-borne pathogens Ehrlichia, Hepatozoon, and Babesia co-infection in owned dogs in Central Thailand
Source: Front Vet Sci. 2024 Apr 2;11:1341254. doi: 10.3389/fvets.2024.1341254 (PMC11019389; doi:10.3389/fvets.2024.1341254)
Supplement: Supplementary file 1 [file Table_1.DOCX]

**Supplementary Table 1** Infection rate of tick-borne pathogens: *Ehrlichia*, *Hepatozoon*, *Babesia*, and co-infection by sex (A) and age (B)

A.

|  | **Male** | | **Female** | | ***p*-value** |
| --- | --- | --- | --- | --- | --- |
|  | **No.** | **%** | **No.** | **%** |  |
| **Ehrlichia** | 41/299 | 13.7 | 28/266 | 10.5 | 0.303 |
| **Hepatozoon** | 3/299 | 1.0 | 5/266 | 1.9 | 0.485 |
| **Babesia** | 8/299 | 2.7 | 6/266 | 2.3 | 0.793 |
| **Co-infection** | 8/299 | 2.7 | 9/266 | 3.4 | 0.632 |
| **Overall** | 60/299 | 20.1 | 48/266 | 18.0 | 0.592 |

B.

|  | **<1 year** | | **1-3 year** | | - 1. **year** | | **7-10 year** | | ***>*10 year** | | ***p*-value** |
| --- | --- | --- | --- | --- | --- | --- | --- | --- | --- | --- | --- |
|  | **No.** | **%** | **No.** | **%** | **No.** | **%** | **No.** | **%** | **No.** | **%** |  |
| **Ehrlichia** | 5/32 | 15.6 | 10/74 | 13.5 | 15/86 | 17.4 | 21/177 | 11.9 | 13/161 | 8.1 | 0.259 |
| **Hepatozoon** | 1/32 | 3.1 | 0/74 | 0.0 | 0/86 | 0.0 | 4/177 | 2.3 | 2/161 | 1.2 | 0.387 |
| **Babesia** | 0/32 | 0.0 | 1/74 | 1.4 | 1/86 | 1.2 | 4/177 | 2.3 | 6/161 | 3.7 | 0.550 |
| **Co-infection** | 1/32 | 3.1 | 7/74 | 9.5 | 1/86 | 1.2 | 5/177 | 2.8 | 3/161 | 1.9 | **0.022*** |
| **Overall** | 7/32 | 21.9 | 18/74 | 24.3 | 17/86 | 19.8 | 34/177 | 19.2 | 24/161 | 14.9 | 0.503 |

No. = Number of positive/number of tested

*Indicates the different levels of significance as *p* <0.05.
