# Supplementary material for: Tick-borne pathogens Ehrlichia, Hepatozoon, and Babesia co-infection in owned dogs in Central Thailand
Source: Front Vet Sci. 2024 Apr 2;11:1341254. doi: 10.3389/fvets.2024.1341254 (PMC11019389; doi:10.3389/fvets.2024.1341254)
Supplement: Supplementary file 2 [file Table_2.DOCX]

**Supplementary Table 2** Infection rate of tick-borne pathogens: *Ehrlichia, Hepatozoon, Babesia*, and Co-infection with rainfall intensity by month

| **Month** | **E** | **H** | **B** | **E,H** | **E,B** | **H,B** | **E,H,B** | **Neg** | **Infection rate** | | | | **Rainfall**  **Intensity**  **(mm.)** |
| --- | --- | --- | --- | --- | --- | --- | --- | --- | --- | --- | --- | --- | --- |
|  |  |  |  |  |  |  |  |  | **Overall** | | **Co-infection** | |  |
|  |  |  |  |  |  |  |  |  | **No.** | **%** | **No.** | **%** |  |
| **Jan** | 3 | 0 | 1 | 0 | 0 | 0 | 0 | 36 | 4/40 | 10.0 | 0/40 | 0.0 | 20.7 |
| **Feb** | 4 | 0 | 0 | 0 | 0 | 0 | 0 | 25 | 4/29 | 13.8 | 0/29 | 0.0 | 62.4 |
| **Mar** | 3 | 2 | 0 | 0 | 0 | 0 | 0 | 37 | 5/42 | 11.9 | 0/42 | 0.0 | 82.8 |
| **Apr** | 4 | 0 | 0 | 0 | 0 | 0 | 0 | 29 | 4/33 | 12.1 | 0/33 | 0.0 | 78.7 |
| **May** | 4 | 0 | 3 | 0 | 0 | 0 | 0 | 36 | 7/43 | 16.3 | 0/43 | 0.0 | 191.3 |
| **Jun** | 4 | 0 | 1 | 0 | 0 | 0 | 2 | 32 | 7/39 | 17.9 | 2/39 | 5.1 | 156.2 |
| **Jul** | 9 | 0 | 1 | 0 | 1 | 0 | 0 | 57 | 11/68 | 16.2 | 1/68 | 1.5 | 192.5 |
| **Aug** | 7 | 2 | 1 | 3 | 2 | 0 | 0 | 59 | 15/74 | 20.3 | 5/74 | 6.8 | 225.7 |
| **Sep** | 8 | 2 | 1 | 0 | 0 | 0 | 0 | 44 | 11/55 | 20.0 | 0/55 | 0.0 | 342.7 |
| **Oct** | 7 | 1 | 0 | 0 | 2 | 0 | 0 | 28 | 10/38 | 26.3 | 2/38 | 5.3 | 206.2 |
| **Nov** | 7 | 0 | 6 | 0 | 2 | 1 | 1 | 36 | 17/53 | 32.1 | 4/53 | 7.5 | 84.5 |
| **Dec** | 9 | 1 | 0 | 0 | 1 | 2 | 0 | 38 | 13/51 | 25.5 | 3/51 | 5.9 | 4.0 |
| **Total** | 69 | 8 | 14 | 3 | 8 | 3 | 3 | 457 | 108/565 | 19.1 | 17/565 | 3.0 |  |

E = *Ehrlichia*, H = *Hepatozoon*, B =*Babesia*

No. = Number of positive/number of tested
