# Supplementary material for: Tick-borne pathogens Ehrlichia, Hepatozoon, and Babesia co-infection in owned dogs in Central Thailand
Source: Front Vet Sci. 2024 Apr 2;11:1341254. doi: 10.3389/fvets.2024.1341254 (PMC11019389; doi:10.3389/fvets.2024.1341254)
Supplement: Supplementary file 3 [file Table_3.DOCX]

**Supplementary Table 3** Hematological alterations in dogs infected with *Ehrlichia, Hepatozoon, Babesia*, and Co-infection*.*

| **Hematological alterations** | **E.** | | **H** | | **B** | | **E+H** | | **E+B** | | **H+B** | | **E+H+B** | |
| --- | --- | --- | --- | --- | --- | --- | --- | --- | --- | --- | --- | --- | --- | --- |
|  | No. | % | No. | % | No. | % | No. | % | No. | % | No. | % | No. | % |
| **Thrombocytopenia** | 57/66 | 86.4 | 5/7 | 71.4 | 10/11 | 90.9 | 2/3 | 66.7 | 8/8 | 100.0 | 3/3 | 100.0 | 3/3 | 100.0 |
| **Eosinopenia** | 45/66 | 68.2 | 3/7 | 42.9 | 9/11 | 81.8 | 3/3 | 100.0 | 6/8 | 75.0 | 2/3 | 66.7 | 3/3 | 100.0 |
| **Hyperproteinemia** | 43/66 | 65.2 | 4/7 | 57.1 | 5/11 | 45.5 | 1/3 | 33.3 | 4/8 | 50.0 | 1/3 | 33.3 | 2/3 | 66.7 |
| **Anemia** | 23/66 | 34.8 | 0/7 | 0.0 | 4/11 | 36.4 | 1/3 | 33.3 | 3/8 | 37.5 | 1/3 | 33.3 | 1/3 | 33.3 |
| **Thrombocytopenia+ Eosinopenia** | 40/66 | 60.6 | 3/7 | 42.9 | 8/11 | 72.7 | 2/3 | 66.7 | 6/8 | 75.0 | 1/3 | 33.3 | 3/3 | 100.0 |
| **Thrombocytopenia+ Eosinopenia+ Hyperproteinemia** | 29/66 | 43.9 | 2/7 | 28.6 | 2/11 | 18.2 | 1/3 | 33.3 | 3/8 | 37.5 | 1/3 | 33.3 | 2/3 | 66.7 |
| **Thrombocytopenia+ Eosinopenia+ Hyperproteinemia+**  **Anemia** | 10/66 | 15.2 | 0/7 | 0.0 | 1/11 | 9.1 | 1/3 | 33.3 | 3/8 | 37.5 | 1/3 | 33.3 | 1/3 | 33.3 |
| **Leucocytosis** | 11/66 | 16.7 | 0/7 | 0.0 | 3/11 | 27.3 | 1/3 | 33.3 | 0/8 | 0.0 | 1/3 | 33.3 | 3/3 | 100.0 |

E = *Ehrlichia*, H = *Hepatozoon*, B =*Babesia.*

No. = Number of positive/number of tested
